# A Yeast Purification System for Human Translation Initiation Factors eIF2 and eIF2Bε and Their Use in the Diagnosis of CACH/VWM Disease

**DOI:** 10.1371/journal.pone.0053958

**Published:** 2013-01-15

**Authors:** Rogerio A. de Almeida, Anne Fogli, Marina Gaillard, Gert C. Scheper, Odile Boesflug-Tanguy, Graham D. Pavitt

**Affiliations:** 1 Faculty of Life Sciences, The University of Manchester, Manchester, United Kingdom; 2 Laboratoire GReD (Génétique, Reproduction et Développement), Faculté de Médecine, INSERM U1103 CNRS 6293, Clermont-Ferrand, France; 3 Université de Clermont, UFR Médecine, Clermont-Ferrand, France; 4 Centre Hospitalier Universitaire de Clermont-Ferrand, Service de Biochimie Médicale et Biologie Moléculaire, Clermont-Ferrand, France; 5 Department of Pediatrics, VU University Medical Centre, Amsterdam, The Netherlands; 6 INSERM U676, Hopital Robert Debré, Paris, France; 7 Université Paris Diderot, Sorbonne Cité, UMR U676, Paris, France; 8 APHP, Hopital Robert Debré, Pediatric Neurology and Metabolic Diseases, Reference Center for Leukodystrophies, Paris, France; University of British Columbia, Canada

## Abstract

Recessive inherited mutations in any of five subunits of the general protein synthesis factor eIF2B are responsible for a white mater neurodegenerative disease with a large clinical spectrum. The classical form is called Childhood Ataxia with CNS hypomyelination (CACH) or Vanishing White Matter Leukoencephalopathy (VWM). eIF2B-related disorders affect glial cells, despite the fact that eIF2B is a ubiquitous protein that functions as a guanine-nucleotide exchange factor (GEF) for its partner protein eIF2 in the translation initiation process in all eukaryotic cells. Decreased eIF2B activity measured by a GEF assay in patients’ immortalised lymphocytic cells provides a biochemical diagnostic assay but is limited by the availability of eIF2 protein, which is classically purified from a mammalian cell source by column chromatography. Here we describe the generation of a recombinant expression system to produce purified human eIF2 from yeast cells. We demonstrate that human eIF2 can function in yeast cells in place of the equivalent yeast factor. We purify human eIF2 and the C-terminal domain of human eIF2Bε using affinity chromatography from engineered yeast cells and find that both function in a GEF assay: the first demonstration that this human eIF2Bε domain has GEF function. We show that CACH/VWM mutations within this domain reduce its activity. Finally we demonstrate that the recombinant eIF2 functions similarly to eIF2 purified from rat liver in GEF assays with CACH/VWM eIF2B-mutated patient derived lymphocytic cells.

## Introduction

Childhood Ataxia with CNS hypomyelination (CACH) or Vanishing White Matter Leukoencephalopathy (VWM) (OMIM #603896) was described in the 1990s [1], [2]. It is a fatal childhood onset white matter disease with a chronic progressive course exacerbated by acute episodes [3], [4]. Inherited mutations in any of the five genes encoding subunits of the general protein synthesis initiation factor eIF2B (*EIF2B1-5*) cause CACH/VWM [3], [5]. The subsequent description of a large clinical spectrum of the disease from neonatal to adult onset or even asymptomatic forms led to the concept of eIF2B-related disorders that are recognized by peculiar magnetic resonance imaging (MRI) abnormalities [6]. Well over 100 different, mainly missense, mutations have been presently reported [4]. Their consequences on the eIF2B complex have been demonstrated in yeast and in humans [7], [8], [9]. Abnormalities in glial cell development have been suggested by studies of patient samples [10], [11], [12], [13], [14], [15], [16] and studies of a mouse model [17]. One recent suggested explanation is that altered expression of splicing regulatory factors in eIF2B mutated glial cells may cause altered splicing regulation of the important myelin proteins PLP and DM20 [18]. However many aspects of the disease are still not understood and no current therapy is available.

eIF2B is well established as a key regulated general translation initiation factor. It functions as a guanine nucleotide exchange factor (GEF) to accelerate the dissociation of GDP from its substrate eIF2•GDP in the first step of translation initiation to form eIF2•GTP [19]. This complex then binds to initiator methionyl tRNA (Met-tRNA_i_^Met^) to deliver it to 40S ribosomes in a reaction that is stimulated by several other translation factors and is required for each translation initiation event on almost all mRNAs [20], [21]. During the initiation cycle eIF2-bound GTP is hydrolysed to GDP to inactivate eIF2 and reset the system. eIF2B is regulated by cellular stresses facilitating translational control in a wide variety of settings. Most widely studied is the activation of eIF2α kinases that phosphorylate eIF2 on its alpha subunit at Ser51 [22]. This phosphorylation reaction converts eIF2 from substrate to inhibitor of eIF2B. When phosphorylated at Ser51, eIF2 binds with higher affinity to eIF2B, but without undergoing nucleotide exchange [19]. Genetic and biochemical studies using eIF2B from yeast identified mutations in three subunits of eIF2B (eIF2Bαβδ) that disrupt this regulation and define a potential regulatory interface between eIF2 and eIF2B that is critical for regulation [23]. Subsequent experiments verified that equivalent mutations in mammalian eIF2B also disrupt eIF2B regulation confirming conservation of this regulatory mechanism [24], [25].

eIF2B activity is measured by a GEF assay that was first established in the 1980s [26], [27]. eIF2 forms a stable complex with GDP in the presence of physiological concentrations of Mg^2+^ ions. It requires significant amounts of eIF2 protein purified from rat liver, rabbit reticulocytes or mammalian cell lysates by rounds of conventional column chromatography [27], [28]. Pure eIF2 mixed with radiolabelled GDP in the presence of Mg^2+^ acts as a substrate and eIF2B is added along with excess unlabelled nucleotide. eIF2B promotes release of labelled GDP and this is assayed by monitoring the progressive decline in labelled eIF2 captured on protein-binding filters. Two versions of the assay have been used; employing either purified eIF2B proteins or extracts from cultured cells. Both formats of the assay have been useful in defining that the largest subunit of eIF2B (eIF2Bε) is the only one that possesses GEF activity, and that this activity is enhanced by complex formation with the other four subunits. Catalytic activity has been shown for yeast, drosophila and mammalian proteins [29], [30], [31]. In addition experiments using purified yeast proteins showed that the C-terminal ∼200 amino acids contained all the elements necessary for minimal exchange function. This region was termed the catalytic domain εcat [32], [33].

The cell extract format of the assay has been used to assess eIF2B activity in immortalised lymphocytic cells isolated from blood samples of CACH/VWM patients [9], [10], [34]. This provides a biochemical diagnostic assay to complement MRI and genetic analyses. The adoption of this GEF assay for diagnostic purposes is limited by the availability of purified eIF2 protein. We, and others, have previously developed a yeast cell expression system to overexpress and purify epitope tagged yeast eIF2, which was subsequently used for *in vitro* studies [29], [35]. Generating recombinant systems for mammalian eIF2 has proved challenging because eIF2 possesses three different subunits in a 1∶1:1 complex, and because popular expression host cells, including *Escherichia coli*, appear refractory to expressing significant amounts of the gamma subunit. We therefore decided to develop a recombinant yeast cell system to purify active human eIF2 protein.

## Results and Discussion

### Human eIF2 Subunits can Complement the Function of the Equivalent Yeast Gene

**Figure 1:**
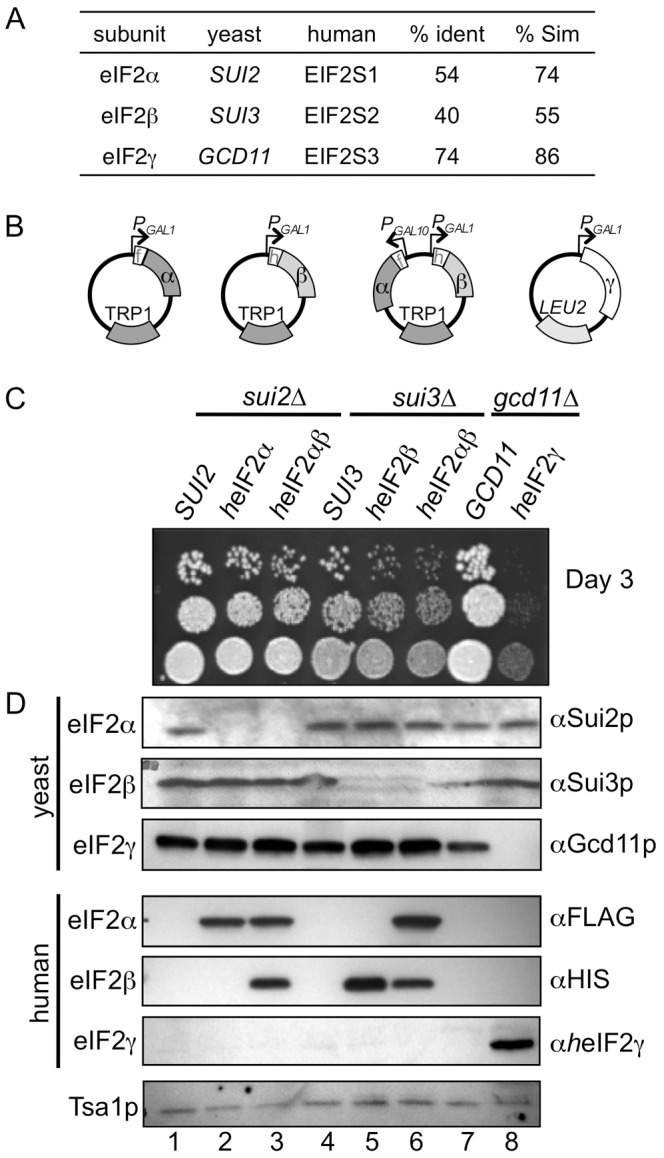
Individual *h*eIF2 cDNAs replace the function of the corresponding yeast gene. A. Table comparing yeast and human eIF2 subunit proteins. B. Cartoon depiction of plasmids pAV1907 (α), pAV1901 (β), pAV1905 (α,β) and pAV1970 (γ), respectively that express the indicated human eIF2 subunits from either *GAL1* or *GAL10* promoters. N-terminal his6 (h) and flag (f) epitope tags are also shown. C. Growth of yeast strains following plasmid shuffling on YPGal medium and D. Western blotting of the same strains using the antibodies indicated. Tsa1p is shown as a loading control. Strains shown in lanes 1–8 are: GP3001, GP5108, GP5109, GP5010, GP5110, GP5111, GP5012, GP5613.

The translation initiation factor eIF2 performs critical roles in the initiation and control of protein synthesis in eukaryotic cells. eIF2 is composed of three non-identical subunits and must interact with GDP, GTP, Met-tRNA_i_^Met^, eIF3, eIF5, eIF2B and 40S ribosomes to perform its functions [35], [36], [37] as well several eIF2α protein kinases for regulation. eIF2 has an archaeal homologue, but is not found in eubacteria [38]. eIF2 subunits are highly conserved between yeast and mammals, including humans (Figure 1A). We decided to develop a yeast system as an expression vehicle for human eIF2 (*h*eIF2). As a first step we obtained cDNA clones and subcloned them into yeast expression vectors, under the control of conditional (galactose carbon source inducible) yeast promoters and bearing short terminal epitope tags (Figure 1B and Materials and Methods). Each yeast eIF2 gene is essential (*SUI2*, *SUI3*, and *GCD11* encoding eIF2α-γ respectively). Strains bearing individual gene deletions covered by a plasmid copy of the yeast gene were used to assess the function of the human expression clones. Each human vector was introduced into its corresponding yeast deletion strain by transformation and plasmid shuffling was used to evict the covering plasmid. We found that plasmids bearing *EIF2S1* encoding *h*eIF2α complemented a *sui2*Δ (Figure 1C, compare lanes 2 and 3 with lane 1) and grew as well as wild type yeast. Similarly plasmids bearing *EIF2S2* encoding *h*eIF2β complemented *sui3*Δ (Figure 1C, compare lanes 5 and 6 with lane 4). However our initial *EIF2S3* plasmids (encoding *h*eIF2γ) could not complement *gcd11*Δ (data not shown). As *EIF2S3* contains a significant number of codons rarely used in yeast we obtained a commercially synthesised yeast-codon-optimized clone and subcloned this into a similar compatible yeast expression plasmid. This could complement the *gcd11*Δ strain, but the resulting strain grew poorly (Figure 1C, lane 8). Western blotting confirmed both deletion of endogenous yeast genes and expression of the corresponding *h*eIF2 subunits (Figure 1D). Because eIF2 functions as a heterotrimer, in these complemented cells heterologous eIF2 complexes should form, each with one human and two yeast subunits.

**Figure 2:**
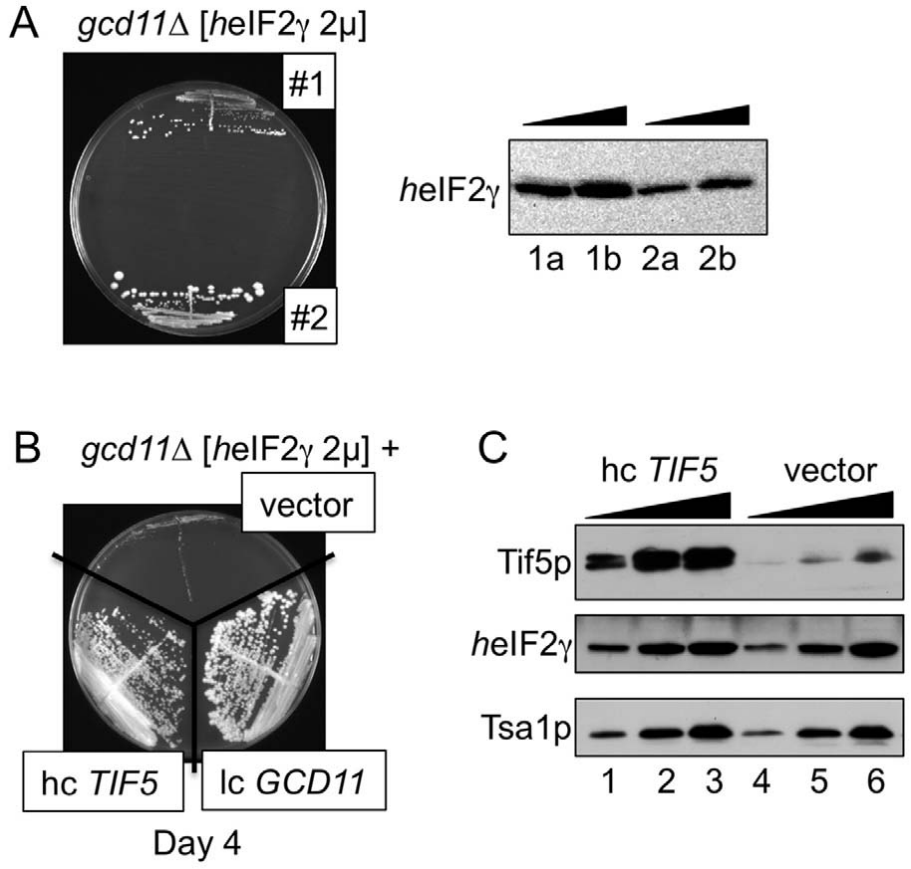
Reducing *h*eIF2γ expression levels or overexpressing eIF5 suppress the slow growth phenotype conferred by *h*eIF2γ overexpression. A. Reciprocal variation in growth and expression level of *h*eIF2γ in 5-FOA resistant colonies (#1 and #2) following transformation of pAV1970 into GP5012. Left, growth on YPGal medium (Day 7). Right, anti-*h*eIF2γ western blot from liquid cultures of the same transformants: lanes a and b 1x and 2x loadings, respectively. B. Transformant #1 (GP5613) was transformed plasmids pRS426, pAV1280 or pAV2015 to make strains GP5744 (vector), GP5755 (*GCD11*) and GP5758 (hc *TIF5*) respectively and grown on YPGal medium (Day 4). C. western blots showing increasing loadings (0.5X, 1X, 2X) of cell extracts from GP5758 (lanes 1–3) and GP5744 (lanes 4–6) with indicated antibodies. Tsa1p is shown as a loading control.

We were concerned by the slow growth of the *EIF2S3* complemented strain (Figure 1C, lane 8), as this may indicate that the codon-optimized cDNA is not fully functional. Several explanations are possible. Firstly, *h*eIF2γ may not be expressed at a high enough level to form sufficient eIF2 complexes for rapid growth. Expression of *h*eIF2γ did not alter the expression levels of the yeast α and β subunits (Figure 1D). We observed that different *EIF2S3* transformants grew at different rates. When the expression level of *h*eIF2γ was examined in a selection of these cells, we consistently found that transformants expressing the highest *h*eIF2γ levels grew more slowly than those with lower expression (Figure 2A and data not shown). These results are therefore more consistent with the idea that the slow-growth phenotype is related to excess levels of *h*eIF2γ. Excess free *h*eIF2γ may bind to and sequester one or more interacting factors into non- or partially functional complexes. This second idea however is unlikely as the slow-growth phenotype is recessive. *h*eIF2γ is only slow growing in the absence of yeast *GCD11* (Figure 2B and data not shown).

Because eIF2γ is the 'core' subunit that binds to both eIF2α and β [38], excess *h*eIF2γ may form some αγ or βγ complexes and thereby reduce the level of full αβγ complexes. If so, then reducing excess *h*eIF2γ may ameliorate complex disruption and improve growth as observed. Alternatively, because eIF2 must interact with many yeast factors including translation factors (eIF2B, eIF5, eIF3) Met-tRNA_i_^Met^ and 40S ribosomes, *h*eIF2γ may not interact effectively with one or more of these. We assessed whether transforming the *h*eIF2γ strain with additional copies of yeast eIF2-interacting factors could complement the growth phenotype by mass action. A panel of high copy plasmids expressing tRNA_i_^Met^ or combinations of subunits of eIF2B, eIF3 or eIF5 was transformed into the *gcd11*Δ strain expressing *h*eIF2γ (see Methods for plasmids assessed). Only excess eIF5 reproducibly suppressed the slow-growth phenotype (Figure 2B and data not shown). Western blotting confirmed that slow-growth suppression in these cells was not caused by reduced *h*eIF2γ expression (Figure 2C). eIF5 interacts with both eIF2β and γ [36] as part of the multifactor complex [39], 43S pre-initiation complex and free eIF2 [40], [41]. It has GTPase acceleration and GDP-dissociation inhibitor functions [42]. While not conclusive, perhaps impaired contact between the hybrid eIF2 and yeast eIF5 affects eIF2 or eIF5 function and that this could be suppressed by mass action. For example excess eIF5 may prevent its premature loss from initiating ribosomes. Alternatively it may stabilise eIF2βγ interactions within the eIF2 complex. Because eIF5 interacts with many initiation factors, several other explanations where the suppressing effect is less direct are also possible to envisage. As the main focus of our study was to generate an expression system to purify active human eIF2, we decided not to investigate these heteromeric yeast-human hybrid complexes further and instead focussed on co-expression of all three human subunits.

### Co-expression of All Three heIF2 Subunits Complements a Triple Yeast eIF2 Gene Deletion Strain

**Figure 3:**
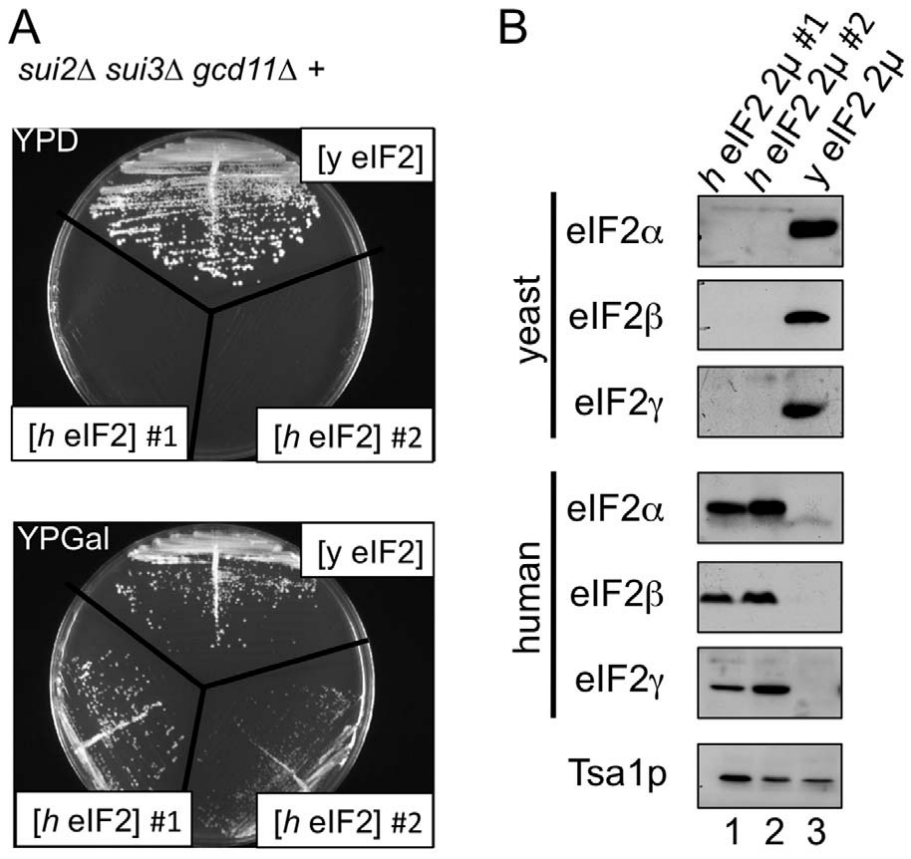
All three *h*eIF2 cDNAs can replace yeast eIF2 in a triple deletion strain. A. Growth of yeast strains GP6124 (*y*eIF2), GP6461 (*h*eIF2 #1), GP6462 (*h*eIF2 #2) on glucose containing YPD medium (represses *h*eIF2 expression; top) and galactose YPGal medium (bottom; *h*eIF2 expression inducing conditions). B. Western blots of cell extracts from the same strains grown in YPGal medium.

Dever and colleagues recently reported construction of a triple eIF2 gene deletion yeast strain complemented by a single plasmid expressing the yeast genes [43]. To assess if co-expression of all three *h*eIF2 subunits could completely replace the yeast factor, we modified this strain to create a *trp1* selectable marker (see Materials and Methods) and transformed in two *h*eIF2 plasmids. One plasmid co-expressed both *h*eIF2αβ and a second plasmid expressed *h*eIF2γ. Plasmid shuffling with FOA generated yeast strains entirely supported by *h*eIF2 (Figure 3A, lower panel). As expected, growth was carbon source dependent. When expression of *h*eIF2 genes from *P_GAL_* promoters was repressed by glucose, *h*eIF2-dependent strains failed to grow (Figure 3A, upper panel). Western blotting confirmed that the yeast genes had been deleted and that higher expression of *h*eIF2 conferred a slower rate of growth (Figure 3B). In conclusion, our *h*eIF2 is functional and can replace the yeast protein *in vivo*.

### Purification of Human eIF2 and Catalytic Domains

**Figure 4:**
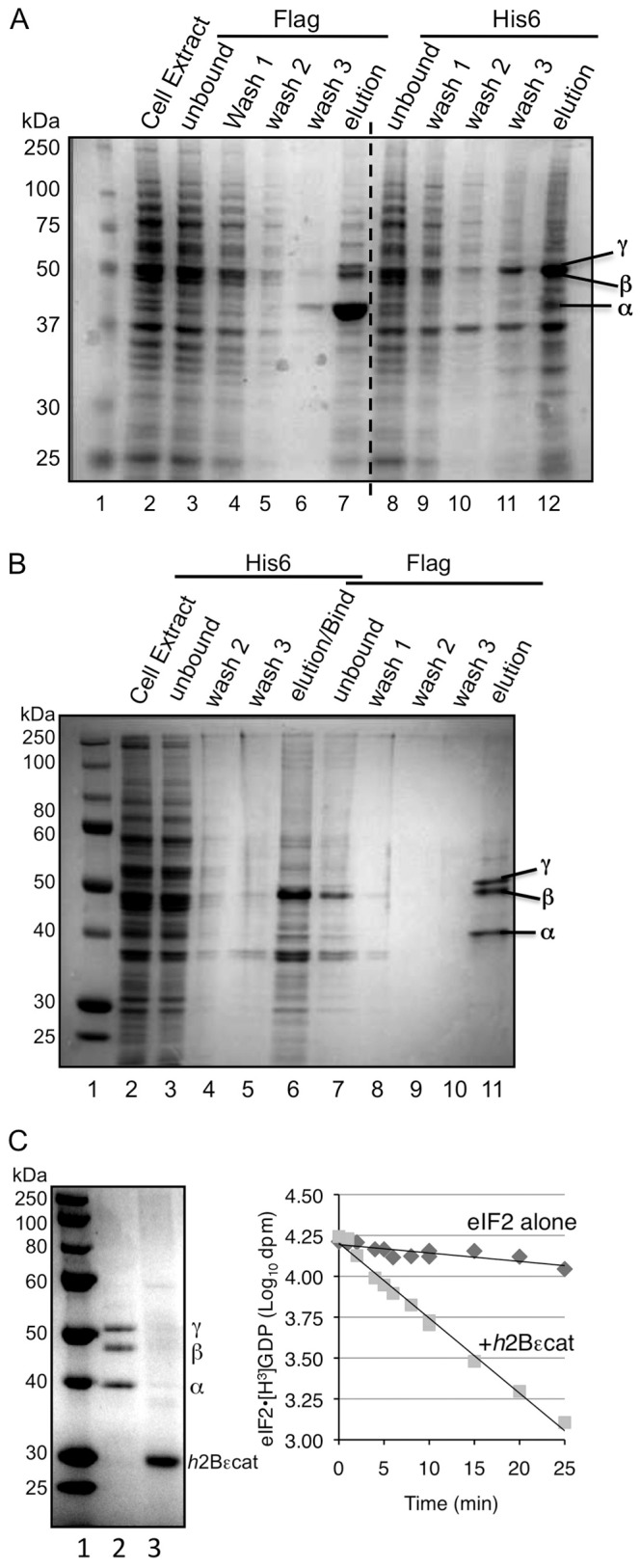
Purification of *h*eIF2, *h*2Bεcat and GEF activity. A. and B.: Coomassie blue stained SDS-PAGE gel summaries of (A) single step partial-purification of *h*eIF2 from yeast strain GP6452 cell extracts (lane 2) using Flag M2 (lanes 3–7) or nickel agarose resins (lanes 8–12) or (B) sequential two-step purification of *h*eIF2 using first nickel agarose (lanes 3–6) and then Flag M2 affinity gel (lanes 7–11) that generated heterotrimeric eIF2. C. Left, coomassie blue stained SDS-PAGE gel of purified human eIF2 (labelled αβγ) and *h*2Bεcat. Right, GEF assays with wild type *h*2Bεcat or eIF2 alone.

Because we appended small epitope tags to each *h*eIF2 subunit, we used affinity chromatography to purify the protein complex. We employed a yeast strain deleted for the only yeast eIF2 kinase *GCN2*. This means that unlike the proteins purified from mammalian sources, our recombinant eIF2 is homogeneously unphosphorylated at the key regulatory site, Ser 51 of the alpha subunit. Purification by a single step using Flag M2 affinity resin to bind *h*eIF2α, or a single nickel agarose step to bind *h*eIF2β was not sufficient to purify *h*eIF2 (Figure 4A). The single step Flag affinity purification recovered a mixture of of αβγ trimers, excess eIF2α and residual contaminating proteins (Figure 4A lanes 2–7). Similarly a single-step his_6_ purification captured a mixture of αβγ trimers and excess eIF2β (Figure 4A, lanes 8–12 and 4B, lanes 2–6). These results are consistent with the known structure of the archaeal homologue of eIF2, aIF2αβγ where both aIF2α and β each separately bind to aIF2γ [38] and with the idea that expression of heIF2γ in our cells is limiting, so that excess free *h*eIF2α and β subunits are formed. We therefore adopted a two-step purification strategy employing nickel agarose, followed by Flag resin (Figure 4B) to purify heterotrimeric eIF2 free from excess α and β subunits (Figure 4B, lane 11). Because eIF2γ expression levels were limiting in our system we transformed in a second plasmid to boost the amounts of the full eIF2 complex. With this strategy we obtained eIF2 that was approximately 90% pure (Figure 4C).

We wished to assess nucleotide binding and exchange with our *h*eIF2 protein. The nucleotide exchange factor eIF2B is specific for eIF2. eIF2B is composed of five distinct subunits. However previous work has shown that the largest subunit alone retains catalytic function and studies in yeast showed that the carboxy terminal domain alone (yeast residues 518–712) is the catalytic domain, termed εcat [32]. Subsequently, deletion of residues 549–596 within human eIF2Bε resulted in a protein that could form eIF2B complexes, but had no GEF activity *in vitro* showing this region is critical for human eIF2B GEF function [8]. In addition X-ray crystallographic structure determination has shown that the human equivalent domain adopts the same stacked, paired α-helical structure as the yeast εcat [33], [44]. We therefore predicted that a construct bearing the equivalent residues of human eIF2Bε, residues 533–721 would comprise the human eIF2B catalytic domain (*h*2Bεcat). A yeast codon-optimized *h*2Bεcat cDNA was synthesized and expressed with tandem Flag and polyhistidine tags from a galactose inducible promoter vector in a suitable yeast strain host and the same purification scheme devised for *h*eIF2 was used (Figure 4C). To assess the functions of purified *h*eIF2 and *h*2Bεcat, we set up a standard nucleotide exchange assay with radiolabelled GDP (see Materials and Methods). In the absence of eIF2B, but in the presence of excess unlabelled GDP, *h*eIF2 bound [^3^H]GDP at 10–30°C with MgCl_2_ from 0.2–2 mM. This indicates that the nucleotide binding function of *h*eIF2 is intact. When *h*2Bεcat was added, this stimulated release of [^3^H]GDP with first order kinetics (Figure 4C). This confirms that nucleotide binding to eIF2 is reversible and that residues 533–721 of human eIF2Bε contains the catalytic domain.

### Catalytic Domain CACH/VWM Mutants have Reduced GEF Activity

As stated in the introduction, mutations in eIF2B cause a genetic disease. Mutations have been found in all five subunits, including several within the catalytic domain. Catalytic domain mutations include missense alleles: P604S [45], I649T [46], E650K [47], M608I [48] and W684S [49] and small deletions: Δ610–613 [9] Δ666–672 [47]. In all patients reported thus far, these alleles occur as compound heterozygotes with other mutations contributing to the disease pathology and the measured eIF2B activity from patient cells. Using a mammalian cell expression system, the Proud laboratory has pioneered *in vitro* biochemical analyses of eIF2B CACH/VWM mutants. Typically HEK293 cells are transformed with vectors overexpressing all five subunits of eIF2B, which are affinity purified and the resulting complexes analysed for complex formation and GEF activity. For example when analysed as part of 5-subunit complex W628R reduced activity to ∼20% [8], I649T to ∼40% and E650K to ∼30% of wild type [49]. The latter two also apparently reduced association with the alpha (and possibly delta) subunits of eIF2B [49] indicating possible reduced complex integrity that could contribute to reduced activity. The Δ610–613 mutant dramatically reduced expression or subunit stability and activity to 25% [9].

**Figure 5:**
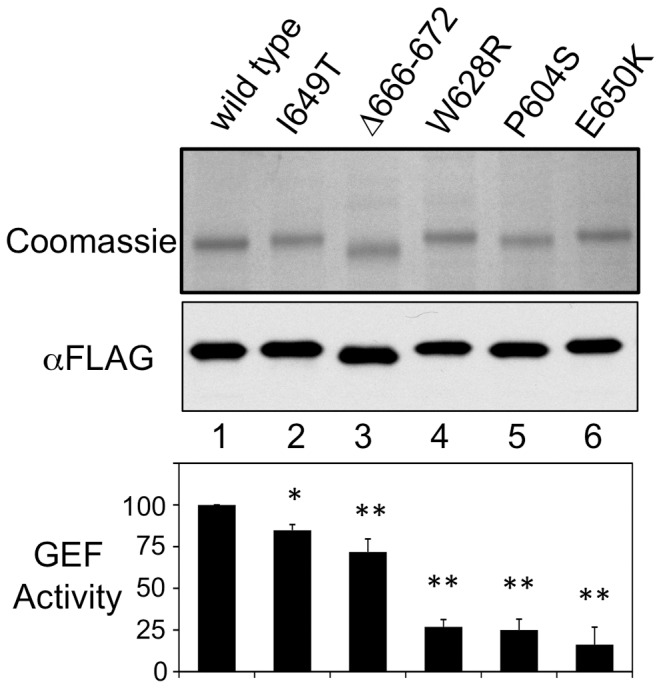
GEF activity of *h*2Bεcat CACH/VWM mutants. Top, purified eIF2B catalytic domains with indicated mutations both coomassie blue stained SDS-PAGE and Flag western blot are shown. Bottom: change in activity normalised to wild type (n = 4). 2 tailed T-test * *p*<0.05, ** *p*≤0.0001.

To further test our recombinant *h*eIF2 and *h*2Bεcat, we introduced several mutations into our *h*2Bεcat expression vector and purified these to homogeneity (Figure 5). We included mutations that had been analysed as part of 5-subunit eIF2B complexes (W628R, I649T and E650K) as well as mutations not analysed previously (P604S and Δ666–672). All mutant forms significantly reduced GEF activity (Figure 5), validating that our *h*eIF2 and *h*2Bεcat behave as expected. It was perhaps surprising that the Δ666–672 mutant had only a modest reduction in activity, when compared with missense mutations that might be predicted to have a smaller impact on the overall structure. Examining the locations of the mutated residues on the human catalytic domain structure [33], [44] suggests that most of the missense mutations affect residues predominantly buried internally within the structure. Macromolecular modelling of the impact of the Δ666–672 mutant, suggests that the normal domain fold can be adopted, but that there is local disruption of part of one α-helix only (data not shown). The affected helix does not contribute to the surface regions of εcat identified previously as critical for direct eIF2 binding and activity [50]. This observation appears to fit with the modest reduction in GEF activity observed for *h*2Bεcat, but does not rule out that it may have a more significant defect *in vivo* in the context of the full five subunit protein complex.

### *h*eIF2 Functions in GEF Assays with Extracts from Patient-derived Cells

Extracts from patient-derived cells have been used as a source of eIF2B to provide biochemical diagnosis for eIF2B related disorders [10], [34]. To assess whether our *h*eIF2 would perform as a substrate in GEF assays with patient cells, we performed assays with lymphoblastoid cell line extracts from 18 CACH/VWM patients and compared the results to those obtained previously with the rat eIF2 substrate classically used for this diagnostic assay (Table 1). Our panel of cell lines included mutations in four of the five eIF2B subunits and those previously shown to have a range of disease severity from severe, early onset to classical/mild forms of disease [34].

**Table 1:** eIF2B GEF activities measured in extracts from lymphoblastoid cell lines from indicated CACH/VWM patents using *h*eIF2 and rat eIF2 substrates.

PatientNumber	Mutatedgene	Gene mutations(protein substitutions)	eIF2B GEF activityusing *h*eIF2 (%)		eIF2B GEF activityusing rat eIF2 (%)*		*h*eIF2/rat eIF2
			Mean	SD	Mean	SD	
949-1	*EIF2B4*	c.728C>T/c.728C>T (Pro243Leu/Pro243Leu)	35.8	3.5	45.6	1.8	0.79
1036-1	*EIF2B5*	c.967C>T/c.1280C>T (Pro323Ser/Pro427Leu)	44.5	9.9	30	7.5	1.48
1838-1	*EIF2B4*	c.818T>C/c.1346C>T (Met273Thr/Thr449Ile)	44.5	2.1	46.8	6.4	0.95
1758-1	*EIF2B5*	c.338G>A/c.947G>A (Arg113His/Arg316Gln)	46.2	1.9	44.8	4.4	1.03
570-2	*EIF2B4*	c.626G>A/c.626G>A (Arg209Gln/Arg209Gln)	49.1	12.8	52	3	0.94
375-2	*EIF2B5*	c.338G>A/c.1948G>A (Arg113His/Glu650Leu)	56.2	3.1	59.4	0.7	0.95
1348-1	*EIF2B5*	c.338G>A/c.338G>A (Arg113His/Arg113His)	56.6	11.6	61.6	1.3	0.92
972-1	*EIF2B5*	c.943C>T/c.271A>G (Arg315Cys/Thr91Ala)	56.6	2.4	60.3	7	0.94
38	*EIF2B2*	c.638A>G/c.638A>G (Glu213Gly/Glu213Gly)	57.5	4	40.3	6.3	1.43
291-1$	*EIF2B5*	c.338G>A/1160A>G (Arg113His/Asp387Gly)	58.6	17.7	41.5	6	1.41
1878-1	*EIF2B5*	c.338G>A/c.338G>A (Arg113His/Arg113His)	59.5	4.5	48.2	5.6	1.23
357-1	*EIF2B5*	c.406C>T/c.1015C>T (Arg136Cys/Arg339Trp)	63.7	17	44.5	4.5	1.43
571-1	*EIF2B5*	c.166T>G/c.944G>A (Phe56Val/Arg315His)	63.9	15.2	40	3	1.60
1963-1	*EIF2B5*	c.338G>A/c.338G>A (Arg113His/Arg113His)	64.4	14	69.2	4.3	0.93
1152-1	*EIF2B5*	c.134C>G/c.134C>G (Ala45Gly/Ala45Gly)	68	1.3	60.8	11.2	1.12
432-1	*EIF2B5*	c.338G>A/c.1884G>A (Arg113His/Trp628X)	86.4	12.4	90.4	1.8	1.04
1240-1	*EIF2B3*	c.604G>A/c.1312C>T (Ala202Thr/Arg438X)	87.2	26	67.7	2	1.29
1074-1	*EIF2B5*	c.338G>A/c.338G>A (Arg113His/Arg113His)	92	5.5	108	11.2	0.85

* Rat eIF2 data from [34].

SD standard deviation.

$ patient previously reported as Arg113His/Arg113His in error [34].

Firstly, we observed a decreased GEF activity (≤87.2%) using the *h*eIF2 substrate for the patient cell lines which exhibited a decreased GEF activity under the previously described diagnostic threshold (77.5%) using rat eIF2 as substrate [34]. The GEF activity values using the *h*eIF2 substrate are marginally higher and show more variability (with a mean SD = 9.1% using *h*eIF2 in comparison to 4.8% using rat eIF2 with a correlation factor between the two assays of r = 0.78. The disease-diagnostic threshold used with this substrate should therefore be revised from 77.5% to 89.9%. Using this new value, the diagnostic impact of the GEF assay is identical to the assay using rat eIF2: the patients with GEF activity <77.5% with rat eIF2 exacerbated also a GEF activity <89.9% with yeast *h*eIF2 (Table 1). There are several possible reasons that may explain the observed minor difference between the two sources of eIF2. Firstly, there are minor sequence variations between the two species. Secondly there may be differences in post-translational modifications between the two preparations. For example, we assume that eIF2 from rat liver will be partially phosphorylated at ser51 by endogenous eIF2 alpha kinases, while *h*eIF2 from yeast is unphosphorylated. Phosphorylated eIF2 inhibits eIF2B reducing the observed activity. Specific mutations in the α, β and δ subunits alter the sensitivity of eIF2B to inhibition by phosphorylated eIF2 [23], [24], [25]. However it is not yet known whether disease-causing mutations alter eIF2B sensitivity to eIF2 phosphorylation.

Cells from two patients tested here (432-1 and 1074-1) exhibited normal GEF activity using the rat eIF2 substrate. Here lower GEF activity was found with the *h*eIF2 substrate, taking patient 432-1 just below the proposed diagnostic threshold (Table 1). A recent report also identified two severe CACH/VWM patients with eIF2B mutations, but no apparent defect in eIF2B activity [49]. However the eIF2B GEF activity for those patients was measured only in primary fibroblasts. Further studies are needed to assess the diagnostic value of measuring eIF2B GEF activity in fibroblasts. Discrepancies have been previously reported between measuring eIF2B defects in immortalised lymphocytic cells compared to primary fibroblasts [51]. In summary, the recombinant *h*eIF2 performs as well as the previously used substrate in this assay and measurement of reduced GEF activity with *h*eIF2 and extracts from immortalised lymphocytic cells is diagnostic for eIF2B-mediated disease.

In conclusion, we describe a strategy for production of heterotrimeric recombinant human eIF2. We show that it can functionally replace yeast eIF2 *in vivo* and demonstrate its utility as a diagnostic tool for measuring the impact of eIF2B mutations that cause human disease. Employing recombinant *h*eIF2 from yeast will remove the need to use animal tissue sources to obtain purified eIF2 for these purposes.

## Methods

### Plasmid Constructions

#### Human eIF2 cDNA expression

Human cDNAs encoding *EIF2S1* (eIF2α) and *EIF2S2* (eIF2β) were cloned with N-terminal Flag or his6 epitope tags respectively into the pBEVY series of plasmids downstream of strong yeast promoters. These constructs failed to express any protein and on resequencing had acquired mutations within the promoter sequences. As the ORFs were intact, they were subcloned into pBEVY-GT, a multicopy yeast expression plasmid bearing divergent promoters from the *GAL1* and *GAL10* genes (*P_GAL1_* and *P_GAL10_*) that we have recently used with success elsewhere [52], [53]. The cDNAs were cloned singly as Kpn1-EcoRI fragments downstream of *P_GAL1_* creating pAV1901 (*EIF2S2*-his_6_) and pAV1907 (*EIF2S1*-Flag). In addition *EIF2S1*-Flag was cloned downstream of *P_GAL10_* as a BamHI-Pst1 fragment in pAV1901 to create pAV1905 (*EIF2S2-*his_6_ and *EIF2S1*-Flag). *EIF2S3* (eIF2γ) cDNA was codon-optimized and synthesized (GeneScript USA Inc.) and subcloned as a Xba1-Sal1 fragment into pBEVY-GL creating pAV1970 and pBEVY-GU creating pAV1974. All constructs were confirmed by DNA sequence analysis. Further details are shown in Table 2.

**Table 2:** Plasmids used or constructed for this study.

Designation	Genes	Source/Construction summary
pAV1228	*SUI3 LEU2 CEN4*	A. Hinnebusch p920
pAV1255	*SUI2 LEU2 CEN4*	A. Hinnebusch p1097
pAV1280	*GCD11 URA3 CEN4*	E. Hannig Ep293
pAV1345	*IMT4 LEU2 2µm*	A. Hinnebusch p1775 [58]
pAV1346	*SUI2 SUI3 URA3 2µm*	A. Hinnebusch p1778 [58]
pAV1348	*GCD11 SUI2 SUI3 URA3 2µm*	A. Hinnebusch p1780 [58]
pAV1427	*P_GAL1_*Flag-His_6_-*GCD6 URA3 leu2d 2µm*	[59]
pAV1428	*GCD1*-Flag_2_-His_6_ *GCD6 URA3 2µm*	[59]
pAV1494	*GCD2 GCD7 GCN3 LEU2 2µm*	[59]
pAV1702	*P_GAL1_ P_GAL10_ 2µm LEU2*	pBEVY-GL dual promoter galactose expression vector [52]
pAV1703	*P_GAL1_ P_GAL10_ 2µm TRP1*	pBEVY-GT dual promoter galactose expression vector [52]
pAV1704	*P_GAL1_ P_GAL10_ 2µm URA3*	pBEVY-GU dual promoter galactose expression vector [52]
pAV1874	*TRP1 P_GPD_EIF2S1*-Flag *P_ADH1_EIF2S2-*His_6_ *2µm*	Epitope tagged human cDNAs amplified and cloned in pBEVY-T Note-Promoter mutations prevent expression.
pAV1901	*TRP1 P_GAL1_EIF2S2-*His_6_ *2µm*	human *EIF2S2-*His_6_ digested from pAV1874 (KpnI/EcoRI) and ligated into pAV1703
pAV1905	*TRP1 P_GAL10_EIF2S1*-Flag *P_GAL1_*His_6_-*EIF2S2 2µm*	human *EIF2S1*-Flag from pAV1874 (BamHI/PstI) ligated into pAV1901
pAV1907	*P_GAL1_EIF2S1*-Flag *2µm TRP1*	human *EIF2S1*-Flag KpnI/EcoRI digested from pAV1874 and ligated into pAV1703
pAV1970	*P_GAL10_*-*EIF2S3 2µm LEU2*	Codon optimised *EIF2S3* cloned into pAV1704 by XbaI/SalI digestion and ligation.
pAV1974	*P_GAL10_*-*EIF2S3 2µm URA3*	Codon optimised *EIF2S3* cloned into pAV1702 by XbaI/SalI digestion and ligation.
pAV2015	*TIF5-Flag URA3 2µm*	K. Asano KAB446
pAV2075	*URA3 P_GAL1_*Flag-His_6_-*EIF2B5cat leu2d 2µm*	Codon optimised commercially synthesized human *EIF2B5* cDNA (codons 533–721 only) cloned MluI/BamHI into pAV1427 removing *GCD6*.
pAV2095	*URA3 P_GAL1_*-Flag-His_6_-*EIF2B5cat*-*I649T leu2d 2µm*	Site directed mutagenesis introduced mutation into *EIF2B5* (533–721) cDNA as described for pAV2075.
pAV2096	*P_GAL1_*-Flag-His_6_-*EIF2B5cat*-Δ*664–671 URA3 leu2d 2µm*	Site directed mutagenesis as above
pAV2097	*P_GAL1_*-Flag-His_6_-*EIF2B5cat*-*W628R URA3 leu2d 2µm*	Site directed mutagenesis as above
pAV2098	*P_GAL1_*-Flag-His_6_-*EIF2B5cat*-*P604S URA3 leu2d 2µm*	Site directed mutagenesis as above
pAV2099	*P_GAL1_*-Flag-His_6_-*EIF2B5cat-E650K URA3 leu2d 2µm*	Site directed mutagenesis as above
pAV2112	*TIF32 NIP1-*His_6_ *LEU2 2µm*	L. Valasek, Prague
pAV2113	P*RT1 TIF35 TIF34 HCR1 URA3 2µm*	L. Valasek, Prague

#### Human eIF2Bε catalytic domain

The *EIF2B5* (eIF2Be) catalytic domain (amino acids 533–721, termed here *h*2Bεcat) was identified by sequence alignment of translated ORF from Genbank (NG_015826) with the yeast *Saccharomyces cerevisie* Gcd6p catalytic domain [32], [33]. The cDNA was codon optimised and custom synthesized (GeneScript USA Inc) with the 3′UTR of *GCD6*. This was subcloned using MluI and BamHI into plasmid pAV1427 in frame with 5' tandem Flag and his_6_ tags downstream of the *P_GAL1_* promoter creating plasmid pAV2075. Quikchange (Agilent) site-directed mutagenesis kit was used to introduce mutations into the GeneScript clone prior to subcloning into pAV1427 to create the plasmids pAV2095-99, each identical to pAV1075 except for the site-directed changes shown in Table 2. All constructs were confirmed by DNA sequence analysis.

### Yeast (*S. cerevisiae*) Genetic Methods

All constructions employed standard methods and yeast media [54]. Transformations used the lithium acetate method. For serial dilution growth assays cells were grown in YPGal liquid medium (2% galactose, 2% raffinose) to logarithmic phase, diluted to A_600_ = 0.1 and 10-fold serially diluted. 2 µl of each dilution was spotted on solid medium and plates were incubated at 30°C. Deletion of *TRP1* in GP5010 and GP5012 (see Table 3) used plasmid pNKY1009 (*trp1*Δ*::hisG-URA3-hisG*) [55]. The *trp1Δ::loxP-hphNT1-loxP* was introduced into GP6124 by PCR amplification using pZC1(pAV2170) [56] as template to disrupt *TRP1* with the *loxP*-hphNT1-l*oxP* cassette. Plasmid shuffling employed 5-fluoro-orotic acid (FOA) [54]. When shuffling plasmids requiring *P_GAL_* expression, cells were grown in YPGal medium prior to shuffling, and FOA medium used 2% galactose in place of glucose. All strains used are summarized in Table 3.

**Table 3:** Yeast Strains Used in this study.

Designation	Genotype	Source/reference
GP3001	*MAT* **a** *leu2-3 leu2-112 sui2*Δ *trp1-*Δ*63 ura3-52* [*SUI2 CEN LEU2*]	Pavitt collection
GP3582	*MAT* **a** *gcd11*Δ*::hisG leu2-3 leu2-112 ino1 ura3-52::HIS4-lacZ* [*URA3 CEN GCD11*]	Pavitt collection, (A. Hinnebusch NIH -F484
GP3889	*MAT*α *gcn2*Δ *leu2-3 leu2-112 pep4::LEU2 trp1-*Δ*63 ura3-52*	Pavitt collection
GP4907	*MAT*α *gcn2*Δ *leu2-3 leu2-112 ino1 sui3*Δ *HIS4-lacZ*::*ura3-52* [*SUI3 CEN LEU2*]	K. Asano, KAY17
GP5010	*trp1*Δ*::hisG* in GP4907	This Study
GP5012	*trp1*Δ*::hisG* in GP3582	This Study
GP5108	[*P_GAL10_EIF2S1-Flag 2µm TRP1]* plasmid shuffle in GP3001	This Study
GP5109	[*P_GAL10_EIF2S1-Flag P_GAL1_EIF2S2-*His_6_ *2µm TRP1]* plasmid shuffle in GP3001	This Study
GP5110	[*P_GAL1_EIF2S2-*His_6_ *2µm TRP1*] plasmid shuffle in GP5010	This Study
GP5111	[*P_GAL10_EIF2S1-Flag P_GAL1_ EIF2S2-*His_6_ *2µm TRP1]* plasmid shuffle in GP5010	This Study
GP5548	GP3889 [*P_GAL1_*Flag-His_6_-*EIF2B5cat URA3 leu2d 2µm*]	This Study
GP5613	[*P_GAL10_EIF2S3 2µm LEU2*] FOA plasmid shuffle in GP5012	This Study
GP5614	[*P_GAL10_EIF2S3 2µm TRP1*] FOA plasmid shuffle in GP5012	This Study
GP5644	GP3889 [*P_GAL1_*-Flag-His_6_-*EIF2B5cat*-*I649T URA3 leu2d 2µm*]	This Study
GP5645	GP3889 [*P_GAL1_*-Flag-His_6_-*EIF2B5cat*-Δ*664-671 URA3 leu2d 2µm*]	This Study
GP5646	GP3889 [*P_GAL1_*-Flag-His_6_-*EIF2B5cat*-*W628R URA3 leu2d 2µm*]	This Study
GP5647	GP3889 [*P_GAL1_*-Flag-His_6_-*EIF2B5cat*-*P604S URA3 leu2d 2µm*]	This Study
GP5648	GP3889 [*P_GAL1_*-Flag-His_6_-*EIF2B5cat-E650K URA3 leu2d 2µm*]	This Study
GP5744	GP5613 [*URA3 2µm*]	This Study
GP5755	GP5613 [*GCD11 CEN URA3*]	This Study
GP5758	GP5613 [*TIF5 2µm URA3*]	This Study
GP6122	*MAT* **a** *gcd11Δ::Nat gcn2Δ::hisG his3Δ0 leu2Δ0 met15Δ0 pep4::HIS3 sui2Δ::hisG sui3Δ::KanMX ura3Δ0*[*GCD11 SUI2 SUI3 URA3 2µm*]	T. Dever J551 [43]
GP6124	*trp1Δ::hphNT1* in GP6122	This Study
GP6452	*GP3889* [*P_GAL10_*-*EIF2S3 2µm LEU2*] [*P_GAL10_EIF2S1-Flag P_GAL1_EIF2S2-*His_6_ *2µm TRP1]* [*P_GAL10_EIF2S3 2µm URA3*]	This Study
GP6461	[*P_GAL10_EIF2S3 2µm LEU2*] [*P_GAL10_EIF2S1-Flag P_GAL1_EIF2S2-*His_6_ *2µm TRP1]* FOA plasmid shuffle in GP6124 (#1)	This Study
GP6462	[*P_GAL10_*-*EIF2S3 2µm LEU2*] [*P_GAL10_EIF2S1-Flag P_GAL1_EIF2S2-*His_6_ *2µm TRP1] FOA* plasmid shuffle in GP6124 (#2)	This Study

To assess complementation of the slow-growth phenotype of strains expressing *EIF2S3* as the sole source of eIF2γ, strains GP5613 or GP5614 were transformed with the following high copy plasmids to alter the levels of the indicated factors: tRNA_i_^Met^ pAV1345 (*IMT4 LEU2*); eIF2B pAV1428 (*GCD1 GCD6 URA3*) and pAV1494 (*GCD2 GCD7 GCN3 LEU2*); eIF5 pAV2015 (*TIF5 URA3*); eIF3 pAV2112 (*TIF31 NIP1 LEU2*) and pAV2113 (*PRT1 TIF35 TIF34 HCR1 URA3*). Only excess eIF5 reproducibly suppressed the slow-growth phenotype. In some experiments other slow-growth suppressing colonies were obtained, but not reproducibly. Except for eIF5, we assume that suppression was caused by altered *EIF2S3* expression similar to that shown in Figure 2A, rather than true suppressive effects of the transformed plasmid. In addition, combinations of overexpressed eIF2 subunits were assessed: pAV1346 (*SUI2 SUI3 URA3*) pAV1348 (*GCD11 SUI2 SUI3 URA3*) or the low copy plasmid pAV1280 (*GCD11 CEN*). Only plasmids bearing *GCD11* suppressed slow-growth.

### Protein Purification

#### eIF2

Strain GP6452 contains plasmids for galactose induced expression of human eIF2 (*h*eIF2) (Table 3). 20 litres SCGal medium containing 2% Galactose +2% Raffinose +0.25% Glucose carbon sources and lacking leucine, tryptophan and uracil was grown to A_600_∼5 in 5 litre flasks. Cells (∼80 g wet weight) were harvested by centrifugation (6000 rpm, 15 mins Beckman JLA8.1000 rotor), washed in ice-cold water and resuspended at 2 ml/g in lysis buffer [100 mM Tris/HCl (pH 8), 500 mM KCl, 5 mM MgCl_2_, 5 mM NaF, 10 mM Immidizole, 7 mM 2-mercaptoethanol, 10% Glycerol, 0.1% Triton X100, protease inhibitor tablet (Roche) and 1 µg/ml pepstatin, 1 µg/ml leupeptin and 1 µg/ml aprotinin and frozen under liquid nitrogen. Cells were lysed using a large cryogenic freezer mill (Spex Certiprep Ltd) and stored at −80°C prior to purification. All subsequent steps were performed at 4°C. Cells were thawed and cell debris removed by centrifugation at 5000 *g* (Sigma 4K15 centrifuge) and the resulting extract was clarified by successive rounds of centrifugation at 22,000 *g*, 30 min, (Heraeus Biofuge Stratos) and 440,000 *g*, 1 hr, (Beckman ultracentrifuge Ti70.1 rotor). Nickel affinity chromatography (Qiagen) was performed in batch mode with rotation for 2 hrs. Resin was collected by low speed centrifugation (2000 rpm), washed four times with Ni Wash buffer (as Lysis buffer, but with 100 mM KCl, 25 mM Immidizole) and eluted (2×1 hr) in Ni Elution buffer (as Ni Wash buffer, but with 500 mM Immidizole). Elutions were combined and dialysed against Flag Wash buffer (as Ni Wash buffer but lacking immidizole), then mixed with 1 ml prewashed Flag M2 agarose resin (Sigma) in batch binding mode for 2 hours. Following three washes in Flag Wash buffer, protein elutions were performed (2×30 min) in Flag Elution buffer [Flag Wash buffer with 0.4 mg/ml 3X Flag peptide (Sigma)]. Finally purified samples were dialysed into storage buffer [30 mM HEPES (pH7.5), 100 mM KCl, 1 mM DTT, 0.1 mM EDTA, 10% glycerol, 0.1% Igepal CA-630] then aliquoted and stored at −80°C. Typically a yield of 2.4 mg eIF2 was purified (Micro-BCA assay, Pierce).

#### eIF2Bε catalytic domain

Strain GP5548 contains plasmid pAV2075 for yeast expression codon-optimized galactose-induced expression of Flag and his_6_ tandem tagged *h*2Bεcat. Strains GP5644-5648 similarly express specific mutant forms of the same protein (Table 3). Our purification scheme was performed as described for human eIF2, except on a smaller scale starting with 8–20 g wet weight cell pellet. Typically 300–500 µg was purified from 20 g starting cell pellet.

### Western Blotting

Extracts from exponentially growing yeast cells were made using glass beads and a FastPrep-24 (MP Biomedicals). Typically 8×A_600_ units of cells were washed and resuspended in 200 µl Laemmli sample buffer, processed for 5×30 seconds at 6 ms^−1^ setting in the FastPrep-24 at 4°C. 20 µl of each sample was resolved on 10 or 12% acrylamide SDS-PAGE, transferred to nitrocellulose membranes and probed with antibodies: Flag M2 (Sigma, 1∶500), His6 (BD Biosciences 51.9000012, 1∶1000) eIF2γ (Abcam AB33207, 1∶1000), Gcd11p (1∶5000; E. Hannig, Texas), Tif5p (1∶1000) [57], Tsa1p (Abcam AB33207, 1∶1000; the human eIF2γ antibody was raised to an epitope shared with yeast Tsa1p: epitope TIKPTVDDD; Tsa1p TIKPTVeDs), Sui2p (1∶1000; T. Dever, NIH), Sui3p (1∶500) [50]. HRP conjugated secondary antibodies (Abcam) and enhanced chemiluminescence detection system (Pierce) were used.

### GEF Assays

#### eIF2B GEF activity measured with purified h2Bεcat

Activity was measured using a standard filter binding assay with eIF2 and radiolabelled GDP. eIF2•[^3^H]GDP binary complexes were formed in binary complex buffer [30 mM HEPES (pH 7.5), 100 mM KCl, 0.1 mM EDTA, 1 mg/ml BSA, 1 mM DTT] with 30 pmol eIF2 and 0.5 µCi [^3^H]GDP (4.5 Ci mmol^−1^) at 20°C for 10 min and stabilized by the addition of 1 mM MgCl_2_. Nucleotide exchange was initiated by the addition of 2 µg *h*2Bεcat and unlabelled GDP (2 nmol). Samples were removed at regular intervals and filtered through nitrocellulose filters, dried and counted by liquid scintillation.

#### eIF2B GEF activity measured in patients cell extracts

An Institutional Review Board of the participating centers (Comité de Protection des Personnes Sud-Est VI, 2009-A00188-49) approved the use of human subjects for this study. A written informed consent was obtained from all patients [34].

Activity measured with extracts from patient lymphoblastoid cell lines (lymphoblasts) as a source of eIF2B was performed with *h*eIF2 as described previously for eIF2 purified from rat liver [7], [25] with the following modifications: the use of 1 µCi [^3^H]GDP (4.5 Ci mmol^−1^) for eIF2•[^3^H]GDP binary complex formation, incubation of this mixture at 30°C for 30 min (instead of 10 min), and the [^3^H]GDP dissociation kinetics was monitored every 5 min (instead of every 2 min: from 0 to 15 min). Such comparative analyses were performed at least in triplicate for cells from 18 patients and matched controls.
